# TRPV4 channel activation induces the transition of venous and arterial endothelial cells toward a pro‐inflammatory phenotype

**DOI:** 10.14814/phy2.14613

**Published:** 2021-01-29

**Authors:** Kathia Beddek, Florent Raffin, Delphine Borgel, François Saller, Diane Riccobono, Régis Bobe, François‐Xavier Boittin

**Affiliations:** ^1^ INSERM Unité Mixte de Recherche‐Santé 1176 Université Paris‐Sud Université Paris‐Saclay Le Kremlin‐Bicêtre France; ^2^ Département des Plateformes unité Analyses Biologiques IRBA (Institut de Recherche Biomédicale des Armées) Brétigny‐sur‐Orge France; ^3^ Département Effets Biologiques des Rayonnements unité de Radiobiologie IRBA (Institut de Recherche Biomédicale des Armées) Brétigny‐sur‐Orge France

**Keywords:** apoptosis, cytoskeleton, endothelial barrier, endothelial cells, ICAM‐1, TRPV4 channel, VE‐Cadherin

## Abstract

The Transient Receptor Potential Vanilloid 4 (TRPV4) of endothelial cells contributes to many important functions including the regulation of Ca^2+^ homeostasis, cell volume, endothelial barrier permeability, and smooth muscle tone. However, its role in the transition of endothelial cells toward a pro‐inflammatory phenotype has not been studied so far. Using both arterial and venous endothelial cells, we first show that the pharmacological activation of TRPV4 channels with GSK1016790A, a potent TRPV4 agonist, triggers robust and sustained Ca^2+^ increases, which are blocked by both TRPV4 antagonists HC067047 and RN9893. TRPV4 activation also triggers the actin cytoskeleton and adherens junction (VE‐Cadherin) rearrangement in both arterial and venous endothelial cells and leads to rapid decreases of trans‐endothelial electrical resistance. In addition to its effect on endothelial barrier integrity, TRPV4 activation selectively increases ICAM‐1 surface expression in arterial and venous endothelial cells, due to the stimulation of ICAM‐1 gene expression through the NF‐κB transcription factor. TRPV4 channel activation also induced apoptosis of venous and arterial endothelial cells, while TRPV4 blockade reduced apoptosis, even in the absence of TRPV4 activation. As altered barrier integrity, increased adhesion molecule expression and apoptosis are hallmarks of the pro‐inflammatory state of endothelial cells, our results indicate that TRPV4 channel activity can induce the transition of both venous and arterial endothelial cells toward a pro‐inflammatory phenotype.

## Graphical abstract

Using both arterial and venous endothelial cells, this paper shows that TRPV4 channel activity can induce the transition of both venous and arterial endothelial cells toward a pro‐inflammatory phenotype, as evidenced by altered barrier integrity, increased adhesion molecule expression, and apoptosis.

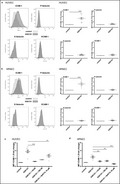

## 1 INTRODUCTION

Endothelial cells which line blood vessels are involved in many important functions including the regulation of vascular tone, vascular permeability, coagulation, inflammation, and angiogenesis. In healthy vessels, endothelial cells inhibit leukocyte adhesion, platelet aggregation and exhibit anti‐inflammatory, anti‐coagulant, and anti‐oxidant properties. Endothelial dysfunction, defined as an impairment of major endothelial cell functions such as vascular tone regulation, anti‐thrombogenic and anti‐inflammatory properties, is a major feature involved in many cardiovascular diseases such as atherosclerosis, heart failure, hypertension, thrombosis, and diabetes (Deanfield et al., 2007; Kwan et al., 2007; Liao, 2013; Sena et al., 2013). Endothelial dysfunction has often been described as an activated state of endothelial cells, due to marked pro‐inflammatory and pro‐coagulant properties. A common hallmark of endothelial dysfunction observed in many diseases is an increased expression of adhesion molecules such as ICAM‐1, VCAM‐1, P‐, and E‐selectin that may facilitate lymphocyte adhesion to activated endothelium (Deanfield et al., 2007; Kwan et al., 2007; Liao, 2013; Sena et al., 2013). Endothelial dysfunction also includes reduced nitric oxide (NO) bioavailability and increased endothelial permeability, resulting in white blood cell infiltration into tissues and edema formation (Deanfield et al., 2007; Kwan et al., 2007; Liao, 2013; Sena et al., 2013). Last, apoptosis of endothelial cells may also be considered as an endothelial dysfunction involved in inflammatory disease as it may result in vascular leak, coagulation, and inflammation, as apoptotic endothelial cells become pro‐adhesive for platelets and leukocytes (Winn & Harlan, 2005).

Endothelial cells express many subtypes of Transient Receptor Potential (TRP) channels and 11 of them have been shown to control important endothelial functions including endothelium‐dependent vasodilation, endothelial permeability, vessel formation, and inflammation (Thakore & Earley, 2019). Several members of this family are, indeed, highly Ca^2+^ permeant and can, therefore, control endothelial cell functions through the regulation of cytosolic Ca^2+^ concentration (Earley & Brayden, 2015; White et al., 2016). Among all the TRP channels found in endothelial cells, Transient Receptor Potential Vanilloid 4 (TRPV4) channels are highly expressed in most if not all endothelial cells (Earley & Brayden, 2015; White et al., 2016).

TRPV4 channels can be activated by a wide range of stimuli like mechanical deformation, osmotic stress/cell swelling, shear stress, low pH, and mild temperatures (Du et al., 2014; Vriens et al., 2004; White et al., 2016). Moreover, these channels are activated by arachidonic acid metabolites (endocannabinoid) produced by the Cytochrome P450 epoxygenase pathway, such as 11,12‐epoxyeicosatrienoic acid (Loot et al., 2008; Vriens et al., 2005; Watanabe et al., 2003). These arachidonic derivatives represent endogenous TRPV4 channel activators, but the channel can also be activated using the phorbol derivative 4α‐Phorbol 12,13‐didecanoate or more recent potent agonists (RN‐1747, GSK1016790A) (White et al., 2016).

TRPV4 activation has been demonstrated to exert profound effects on endothelial cell functions. TRPV4 channels of endothelial cells have a prominent role in the endothelium‐dependent control of smooth muscle tone as TRPV4‐induced Ca^2+^ entry can trigger vasodilation through the stimulation of NO release and/or Ca^2+^‐activated potassium channels (Hill‐Eubanks et al., 2014; Mendoza et al., 2010; Naik & Walker, 2018; Simonsen et al., 2017; Sonkusare et al., 2012). Ca^2+^ entry through TRPV4 channels has also been shown to increase endothelial barrier permeability, resulting in vascular leakage, edema, tissue hemorrhage, and circulatory collapse (Alvarez et al., 2006; Simonsen et al., 2017; Suresh et al., 2015; Townsley et al., 2006; Watanabe et al., 2008; Willette et al., 2008; Wu et al., 2009; Yin et al., 2008; Zhao et al., 2018). This effect may be explained by Ca^2+^‐dependent cytoskeletal rearrangement, alteration of tight, and adherens junctions (e.g., ZO1, VE‐cadherin, Beta‐catenin…) and subsequent opening of endothelial junctions between endothelial cells (Jie, et al., 2015; Matsumoto et al., 2018; Phuong et al., 2017; Zhao et al., 2018). Ca^2+^ entry through TRPV4 channels has finally been shown to be involved in the regulation of gene expression, including endothelial NO synthase (eNOS) and the adhesion molecule P‐selectin (Bihari et al., 2017; Pu et al., 2015).

Here, we investigated possible links between TRPV4 activation and development of pro‐inflammatory properties of endothelial cells. We show that direct TRPV4 activation with a selective agonist (GSK1016790A) triggers the transition of both venous and arterial endothelial cells toward a pro‐inflammatory phenotype, as evidenced by altered barrier integrity, increased membrane expression levels of ICAM‐1 and apoptosis/necrosis that can be prevented in presence of the pharmacological inhibition of TRPV4. Additionally, the pharmacological inhibition of TRPV4 also appears to significantly reduce the basal apoptosis of unstimulated endothelial cells. Altogether, these findings highlight the interest of the TRPV4 blockade to protect endothelial cells from apoptosis/necrosis and also to prevent the development of an inflammatory state of the endothelium.

## 2 MATERIALS AND METHODS

### 2.1 Materials

The TRPV4 agonist GSK1016790A and TRPV4 antagonist HC067047 were purchased from Abcam (Cambridge, UK). The TRPV4 agonist RN1747, the TRPV4 antagonist RN9893, and the NF‐κB inhibitors Honokiol and Ro106‐9920 were purchased from Tocris (Bristol, UK). FITC‐conjugated mouse anti‐CD144 (VE‐Cadherin) antibody (clone 55‐7H1, cat no 580411) was purchased from BD Pharmingen and the Phalloidin 547 (FP‐AZ0330) was purchased from FluoProbes®, Interchim, (Montluçon, France). For immunostainings, we purchased the mouse anti‐human ICAM‐1 antibody (MA5407, clone 1A29) from Invitrogen (Paris, France) and the polyclonal rabbit anti‐TRPV4 antibody (SAB2104243) from Sigma Aldrich (St Louis, MO, USA). Secondary antibodies were purchased from Life Technologies (donkey anti‐mouse Alexa Fluor 647 (cat n^o^ A31571) and goat anti‐rabbit Alexa Fluor 555 (Cat n^o^ A21428)). DAPI Fluoromount‐G^TM^ mounting medium (cat no 0100‐20) was purchased from Southern Biotech (Birmingham, AL 35209, USA). For flow cytometry analysis, we used BB515 mouse anti‐human CD54 (ICAM‐1, cat no 564685), BUV737 mouse anti‐human CD106 (VCAM‐1, cat no 565418), PE mouse anti‐human CD62P (P‐Selectin, cat no 555524), APC mouse anti‐human CD62E (E‐Selectin, cat no 551144), and a FITC Annexin V apoptosis detection kit from BD Biosciences. For western blot analysis, we purchased the anti‐ICAM‐1 (H4, cat n° sc‐390483) and anti‐β‐actin (C4, cat n° sc‐47778) from Santa Cruz Biotechnology. BAPTA‐AM was purchased from Sigma Aldrich (St Louis, MO, USA).

### 2.2 Cell culture

Human pulmonary artery endothelial cells (HPAECs) were purchased from PromoCell (Switzerland). HPAECs were cultured in T25 flasks, 6‐well plates or on glass coverslips coated with attachment factor (Gibco), and allowed to grow and reach confluence in endothelial cell growth medium 2 (PromoCell, Switzerland) containing endothelial cell growth medium supplement 2 (PromoCell, Switzerland) and Gentamicin/amphotericin (Gibco).

Human umbilical vein endothelial cells (HUVECs) were purchased from Gibco. HUVECs were cultured in T25 flasks, 6‐well plates or on glass coverslips coated with attachment factor (Gibco), and allowed to grow and reach confluence in medium 200 containing low serum growth supplement and Gentamicin/amphotericin (Gibco).

Cells from passage 3 to 6 were used for all experiments.

### 2.3 Intracellular Ca^2+^ measurements

HPAECs and HUVECs were grown on glass coverslips before been incubated at room temperature with the Ca^2+^‐sensitive dye Fura‐2 a.m. (1 µM) for 45 min in HEPES buffer in presence of Ca^2+^ (CaCl_2_ 1.6 mM). Ca^2+^ influx induced by 30 nM GSK1016790A (GSK101) was analyzed in the presence or not of TRPV4 inhibitors; 1µM HC067047 (HC06) or 10 µM RN9893 (RN98). 510 nm fluorescence emissions in response to 340 and 380 nm excitation were immediately recorded by an epifluorescence microscope (Nikon Eclipse TE2000‐U) and a black and white CCD camera (CoolSNAP HQ Photometrics, Tucson, USA) at 5 s intervals using the Metamorph software. Changes in Ca^2+^ signal intensity were calculated as the ratios of fluorescence measured at 340 and 380 nm and were normalized over basal level measure before cell activation and the area below the curve for 2 min after agonist addition was chosen as an indicator of the Ca^2+^ response.

### 2.4 Immunostaining

HPAECs and HUVECs were seeded on glass coverslips coated with attachment factor (Gibco) and grown until confluence. Cells were first preincubated with medium (with or without 1 µM HC06) for 10 min before a 24 hr exposition to GSK101 (30 nM) at 37°C and 5% CO_2_. Brightfield pictures were acquired before and after GSK101 treatment. Cells were kindly washed with DPBS and fixed with paraformaldehyde 2% for 15 min at room temperature (RT). Washed cells were permeabilized with Triton X‐100 (0.2%) for 3 min and blocked using 5% bovine serum albumin in DPBS for 1 hr at RT.

Cells were labeled either with FITC‐conjugated mouse anti‐CD144 (VE‐Cadherin) antibody (1:5) and Phalloidin 547 (1:250) for 45 min at RT or incubated with primary antibodies: a mouse anti‐human ICAM‐1 antibody (1:200) and a polyclonal rabbit anti‐TRPV4 antibody (1:200) for 1 hr at RT. Cells were washed three times and stained with Alexa Fluor 488 phalloidin and appropriate secondary antibodies (donkey anti‐mouse Alexa Fluor 647 or goat anti‐rabbit Alexa Fluor 555) for 1 hr at RT. The specificity of these secondary antibodies was tested without primary antibody as a negative control. After washing, coverslips were mounted on glass slides using DAPI Fluoromount‐G^TM^. Except for TRPV4 staining, images were captured with an OLYMPUS IX2‐SLP microscope equipped with a Hamamatsu Orca‐Flash4.0 V2 camera and NIS Elements Nikon software (version 4.51.00). The acquisition settings (Led power, exposition time) were kept the same for all conditions (treated and non‐treated cells) in each independent experiment. Background corrections were then performed using the optimal LUTs settings of control images to treat all other images from different conditions.

To address VE‐Cadherin and F‐Actin delocalization, analysis was performed using Image J 1.52a software. Lines passing through the nucleus of cells allowed to plot fluorescence profiles and then to measure plasma membrane and intracellular fluorescence intensity for F‐Actin or VE‐Cadherin. In three independent experiments, plot profiles of 10 representative cells allowed to calculate means plasma membrane and intracellular fluorescence intensity in each condition (control, GSK101‐treated cells, GSK101‐treated cells preincubated with HC06 and HC06‐treated cells). Intensities measured in control were arbitrarily taken as 100%. For GSK101‐treated cells, GSK101‐treated cells preincubated with HC06 and HC06‐treated cells, intensities were expressed as % of control.

Selected cells were of similar size and were connected to other cells. In contrast, cells in division and isolated ones were excluded. In order to choose an area for measurements, images were first overall observed, to ensure for staining homogeneity. Representative area was then chosen to select cells corresponding to the criteria mentioned before.

For TRPV4 staining, acquisitions were performed using a NIKON ELIPSE E600 microscope equipped with a PixeLink PL‐B955F camera. Camera settings were kept the same for HUVECs and HPAECs. To quantify fluorescence intensity from TRPV4 stainings, Image J 1.52a software was used to reduce the background by setting a threshold for all images (at least 11 images) and measure the fluorescence (limited to the threshold zone of TRPV4 labeling). To normalize, the mean ratio of mean fluorescence to the surface area was first calculated for each experiment before statistical analysis to compare TRPV4 expression in HUVECs and HPAECs.

### 2.5 Western blot analysis

Confluent endothelial cells cultured in 6‐well plates were detached using detach kit 125 (PromoCell), centrifuged, and placed in ice‐cold T‐PER (Tissue Protein Extraction Reagent, Thermo Fisher scientific) containing a protease inhibitor cocktail (Calbiochem, 1/200). Proteins were then extracted and solubilized using mini grinders (Bio‐Rad). For western blot analysis, samples were mixed with 4x Laemmli buffer (Bio‐Rad) containing 10% mercaptoethanol, boiled (95°C for 5 min), and loaded on 4%–15% polyacrylamide gels (Mini‐Protean TGX, Bio‐Rad). To assess sample protein molecular mass, molecular weight standards (Bio‐Rad) were loaded on gels. Endothelial cell samples were then run for 35 min for optimal protein separation. A Trans‐Blot Turbo Transfer system (Bio‐Rad) was used to transfer separated proteins on Polyvinylidene Fluoride (PVDF) membranes. PVDF membranes were saturated for 30 min in PBS containing 0.05% Tween 20 and 5% nonfat dry milk. After saturation was completed, PVDF membranes were incubated overnight at 4°C with primary antibodies diluted in PBS containing 0.05% Tween 20 and 5% nonfat dry milk (mouse anti‐ICAM‐1 antibody (Santa Cruz Biotechnology) 1:100). Membranes were then rinsed with PBS containing 0.05% Tween 20 and incubated for 1 hr at room temperature with a horseradish peroxidase‐conjugated anti‐mouse antibody (Santa Cruz Biotechnology) diluted in PBS containing 0.05% Tween 20 and 5% nonfat dry milk. After extensive membrane wash, protein bands were revealed using a chemiluminescent substrate (Clarity Western ECL, Bio‐Rad). An Anti‐β‐actin antibody (Santa Cruz Biotechnology) was used to control protein loading. Bands on membranes were observed using a CCD camera‐coupled gel imager (Bio‐Rad ChemiDoc). Image J software was used to perform densitometric analysis.

### 2.6 Apoptosis assay

Detection of apoptotic cells was performed using a FITC Annexin V apoptosis detection kit (BD Biosciences). Endothelial cells were cultured in 6‐well plates. Once 80% confluency was reached, cells were treated or not with the TRPV4 agonist GSK101 for 24 hr. To investigate the specificity of TRPV4 stimulation with GSK101, cells were incubated with the TRPV4 antagonists HC06 or RN98 10 min before stimulation with GSK101. After 24 hr, endothelial cells were detached using detach kit 125, washed twice with cold PBS, and resuspended in 100 μl of binding buffer. Cells were then incubated with FITC Annexin 5 (5 μl) and Propidium iodide (PI, 5 μl) for 15 min at room temperature in the dark. After staining, binding buffer was added to each tube and flow cytometry analysis was performed rapidly using a Becton Dickinson LSR II cytometer. Data were analyzed with FlowJo software, allowing to discriminate viable cells (PI‐, FITC annexin V‐) from cells undergoing early apoptosis (PI‐, FITC annexin V+), apoptosis (PI+, FITC annexin V+) or rather necrosis (PI+, FITC annexin V‐). For each experiment, percentages of viable, early apoptotic, apoptotic, and necrotic cells were then calculated.

### 2.7 Measurements of endothelial cell surface expression of adhesion molecules

Surface expression of ICAM‐1, VCAM‐1, P‐selectin, and E‐selectin was investigated using flow cytometry. Endothelial cells were cultured in 6‐well plates. Once 80% confluency was reached, cells were treated or not with the TRPV4 agonists GSK101 or RN1747 for 24 hr. To investigate the specificity of TRPV4 stimulation with GSK101 or the role of the transcription factor NF‐κB, cells were incubated with TRPV4 antagonists or NF‐κB inhibitors 10 min before stimulation with GSK101. After 24 hr, endothelial cells were detached using detach kit 125, washed with PBS, and fixed in PBS containing 1% paraformaldehyde. After extensive washing, cells were stained (30 min) with a four colors antibody panel composed of BB515 mouse anti‐human CD54 (ICAM‐1), BUV737 mouse anti‐human CD106 (VCAM‐1), PE mouse anti‐human CD62P (P‐selectin), and APC mouse anti‐human CD62E (E‐selectin). All antibodies were from BD Biosciences. Cells were then extensively washed with PBS and flow cytometry analysis was performed using a Becton Dickinson LSR II cytometer. For each experiment, anti‐Mouse Ig, k/Negative control compensation particles set (BD Biosciences) was used to set compensations. Data were analyzed with FlowJo software. Median fluorescence intensity (MFI) values were used to compare cell surface expression of ICAM‐1, VCAM‐1, P‐selectin, and E‐selectin in control and after cell stimulation with TRPV4 agonists, with or without antagonists or pharmacological compounds.

### 2.8 Trans‐endothelial electrical resistance measurements

Cells were seeded on gelatin‐coated E‐plates L8 (ACEA biosciences, 00300600840) at a density of 50,000 cells/well and placed in the RTCA iCELLigence instrument (ACEA Biosciences.Inc, 2XL8) for impedance measurement. HC06 and GSK101 expositions were performed when cells reached confluence and the Cell Index was stabilized. Area under the curve of normalized Cell Index was measured for 1 hr.

### 2.9 Statistical analysis

Statistical significance was evaluated with Student's *t* test or 1‐way ANOVA, using GraphPad Prism software (San Diego, CA).

## 3 RESULTS

### 3.1 TRPV4 expression/localization in HUVEC and HPAEC

**Figure 1:**
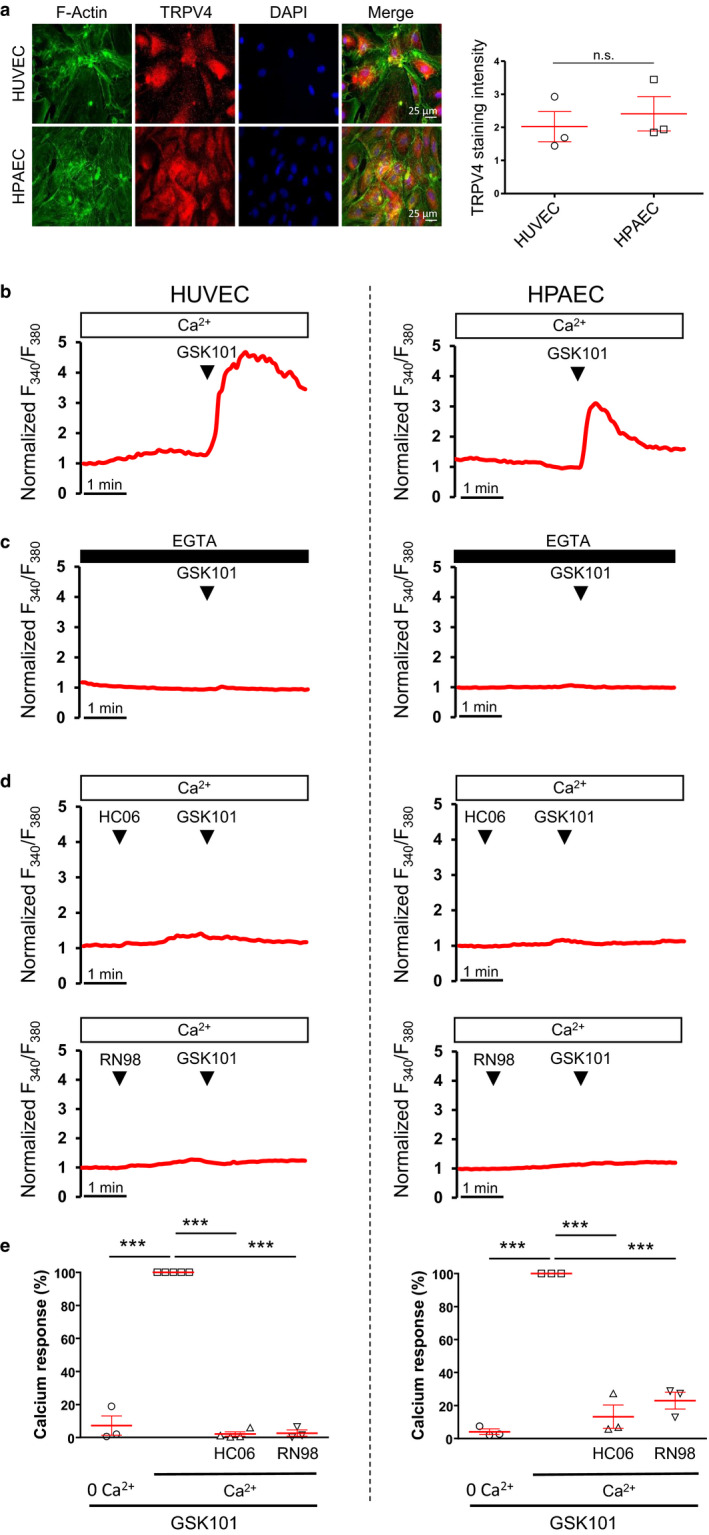
Effect of the TRPV4 agonist GSK1016790A on cytosolic Ca^2+^ levels in confluent HUVECs and HPAECs. (a) Left: Immuno‐localization of TRPV4 channels in confluent HUVEC and HPAEC monolayers. For both HUVECs and HPAECs, TRPV4 and F‐actin immunostainings are represented. Right: Average intensity of TRPV4 staining in HUVECs and HPAECs (see material and methods). Data presented are expressed as means ± *SEM*. Statistical analysis was performed using *t* test (n.s.: not significant). (b) Cytosolic Ca^2+^ increases triggered by bath addition of 30 nM GSK1016790A (GSK101) in HUVECs and HPAECs. (c) Cytosolic Ca^2+^ increases triggered by bath addition of 30 nM GSK101 in HUVECs and HPAECs in the absence of external Ca^2+^ (0 Ca^2+^ + EGTA 100 µM). (d) Cytosolic Ca^2+^ increases triggered by bath addition of 30 nM GSK101 in HUVECs and HPAECs after pre‐treatment with either HC067047 (HC06, 1 µM) or RN9893 (RN98, 10 µM). (e) Averaged Ca^2+^ responses triggered by bath addition of 30 nM GSK101 in HUVECs and HPAECs in control conditions, in cells pre‐treated with either HC06 (1 µM) or RN98 (10 µM), or in the absence of external Ca^2+^. Data presented are representative of three independent experiments and expressed as means ± *SEM*, using 1‐way ANOVA (***p < .001)

Confluent HUVEC and HPAEC endothelial cells were immuno‐stained for the TRPV4 channel. Results from Figure 1a indicate that TRPV4 channels were robustly expressed in both types of endothelial cells. Measurements of TRPV4 staining intensity suggest that TRPV4 expression was not significantly different between HUVECs and HPAECs (Figure 1a).

### 3.2 Pharmacological activation of TRPV4 channels induces Ca^2+^ entry in venous and arterial endothelial cells

Confluent HUVEC and HPAEC endothelial cells were loaded with Fura‐2, allowing the measurement of cytosolic Ca^2+^ concentration. As illustrated in Figure 1b, bath application of the potent TRPV4 channel activator GSK1016790A (GSK101, 30 nM) triggered robust and sustained Ca^2+^ increases in both HUVECs and HPAECs. In both HUVECs and HPAECs, GSK101‐induced Ca^2+^ responses were abolished when Ca^2+^ was removed from the extracellular medium (0 Ca^2+^ + EGTA 100µM, Figure 1c). Moreover, GSK101‐induced Ca^2+^ increases were also blunted when HUVECs or HPAECs had been pre‐incubated with the TRPV4 inhibitors HC067047 (HC06, 1 µM) or RN9893 (RN98, 10 µM) (Figure 1d). Effects of extracellular Ca^2+^ removal and TRPV4 antagonists on GSK101‐induced Ca^2+^ responses are summarized in Figure 1e. Altogether, these results indicate that GSK101‐induced Ca^2+^ responses of both HUVECs and HPAECs are due to Ca^2+^ entry through TRPV4 channels, but not to a non‐specific effect of GSK101.

### 3.3 TRPV4 activation alters endothelial monolayer integrity and triggers the formation of stress fiber and disorganization of adherens junctions (VE‐Cadherin) in venous and arterial endothelial cells

**Figure 2:**
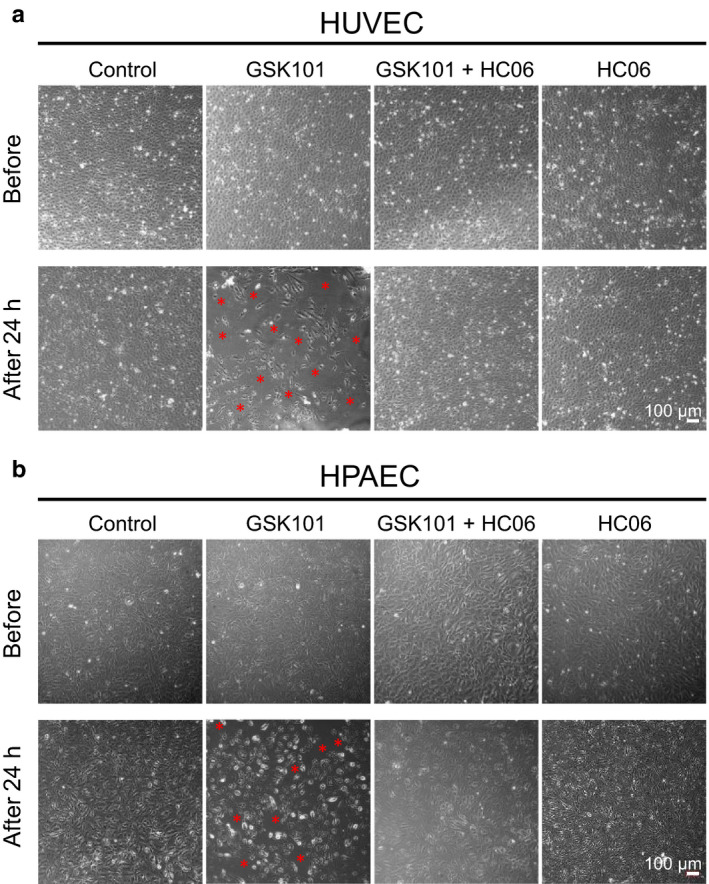
TRPV4 activation with GSK101 alters HUVEC and HPAEC monolayer integrity. (a) Phase contrast images of HUVEC monolayers in control and after the addition of GSK101, with or without HC06 (1 µM), and in the presence of HC06 alone. (b) Phase contrast images of HPAEC monolayers in control and after the addition of GSK101, with or without HC06 (1 µM), and in the presence of HC06 alone. Red stars on images indicate areas with cell loss. For (a and b), images were recorded before the addition of drugs and 24 hr after. Data presented are representative of three experiments

As illustrated in Figure 2a,b, incubation of HUVEC or HPAEC monolayers with 30 nM GSK101 for 24 hr promoted detachment/loss of a fraction of endothelial cells and therefore altered monolayer integrity, as previously reported for HUVECs (Thorneloe et al., 2012). Pre‐incubation of HUVEC or HPAEC monolayers with HC06 (1 µM) completely prevented this effect. Similar deleterious effects of GSK101 were, therefore, observed in HUVEC and HPAEC endothelial monolayers, but detachment/loss of endothelial cells was weaker for arterial endothelial cells.

**Figure 3:**
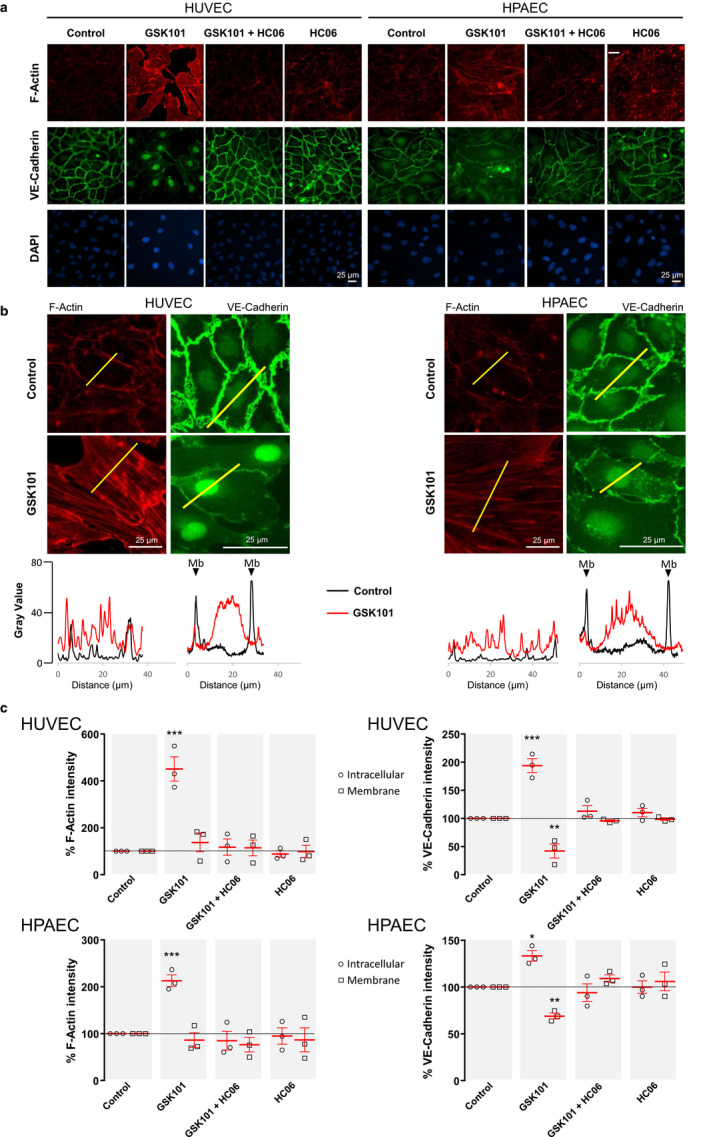
TRPV4 activation with GSK101 alters cytoskeleton (F‐Actin) and adherens junctions (VE‐Cadherin) in HUVECs and HPAECs. (a) Immunostainings of F‐Actin and VE‐Cadherin in HUVEC and HPAEC monolayers in control and 24 hr after the addition of 30 nM GSK101, with or without HC06 (1 µM), and in the presence of HC06 alone. (b) Top: Enlargement of GSK101‐induced effects on F‐Actin and VE‐Cadherin staining in HUVECs and HPAECs. Below: Plot profiles of F‐Actin or VE‐Cadherin fluorescence intensity corresponding to the yellow lines drawn on F‐Actin or VE‐Cadherin fluorescence images in control and after 24 hr stimulation with GSK101 (Mb: Membrane). (c) Graphs represent average membrane and intracellular F‐Actin or VE‐Cadherin fluorescence intensity in control, after 24 hr stimulation with GSK101 (with or without HC06 (1 µM)), and in the presence of HC06 alone. Data presented are the representative of three experiments (see material and methods) and expressed as means ± *SEM* (*** *p* < .001, ** *p* < .01, * *p* < .05)

HUVEC and HPAEC confluent monolayers were stimulated with GSK101 (30 nM) with or without the continuous presence of the TRPV4 inhibitor HC06 (1 µM). To investigate whether TRPV4 stimulation induces cytoskeleton and adherens junction remodeling, endothelial cells were fixed after 24 hr incubation with GSK101 and stained with Phalloidin or anti‐VE‐Cadherin antibody. Results from Figure 3a,b indicate that TRPV4 stimulation with GSK101 for 24 hr induced cytoskeleton rearrangement in both HUVECs and HPAECs. Indeed, GSK101 treatment promoted a marked increase of intracellular F‐Actin and potently stimulated the formation of Phalloidin‐stained F‐Actin stress fibers in both HUVECs and HPAECs (Figure 3b,c). This effect was clearly linked to TRPV4 stimulation as it was abolished when TRPV4 channels had been blocked with HC06 (1 µM, Figure 3a‐c). In contrast, membrane/cortical F‐Actin was not found to be significantly altered on average, although high intensity cortical F‐Actin staining was sometimes observed (Figure 3b,c).

**Figure 4:**
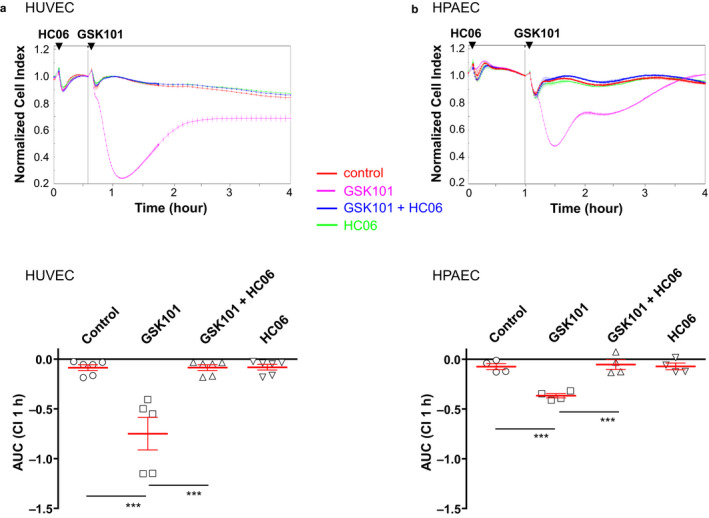
TRPV4 activation with GSK1016790A decreases trans‐endothelial electrical resistance (TER) in HUVEC and HPAEC monolayers. (a) Top: Time course of HUVEC monolayer impedance in control and after the addition of 30 nM GSK101, with or without HC06 (1 µM), and in the presence of HC06 alone. Bottom: Average TER values in control, after the addition of 30 nM GSK101 (with or without HC06 (1 µM)), and after the addition of HC06 alone. (b) Top: Time course of HPAEC monolayer impedance in control and after the addition of 30 nM GSK101, with or without HC06 (1 µM), and in the presence of HC06 alone. Bottom: Average TER values in control, after the addition of 30 nM GSK101 (with or without HC06 (1 µM)), and after the addition of HC06 alone. Data from three independent experiments are expressed as means ± *SEM* (***p < .001)

**Figure 5:**
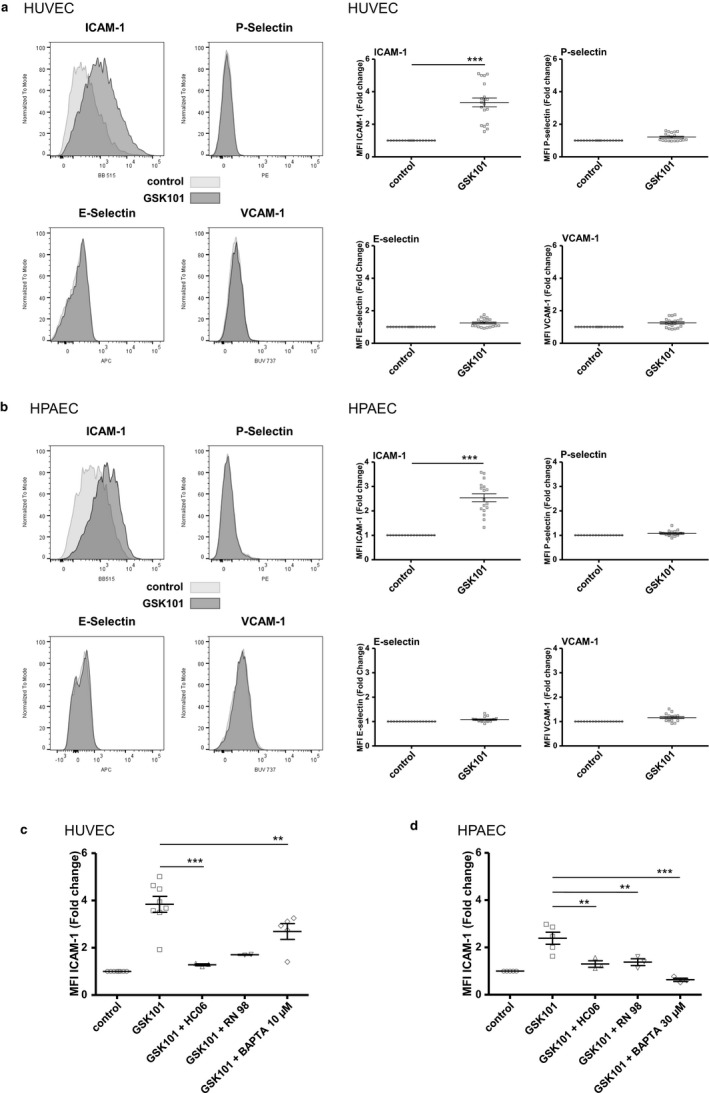
Effect of the TRPV4 agonist GSK101 on the surface expression of ICAM‐1, VCAM‐1, E‐selectin, and P‐selectin in HUVECs and HPAECs. Surface expression of ICAM‐1, VCAM‐1, E‐selectin, and P‐selectin were determined by flow cytometry 24 hr after the addition of 30 nM GSK101 to the culture medium of HUVECs or HPAECs. (a) Left: Representative merged histograms obtained for control HUVECs and HUVECs treated with 30 nM GSK101 for 24 hr. Right: Averaged MFI for ICAM‐1, P‐selectin, E‐selectin, and VCAM‐1 for control HUVECs and for HUVECs treated with 30 nM GSK101 for 24 hr. Data on graphs are expressed as means ± *SEM* and represent the average of 19 experiments. Statistical analysis was performed using *t*‐test (***p < .001). (b) Left: Representative merged histograms obtained for control HPAECs and HPAECs treated with 30 nM GSK101 for 24 hr. Right: Averaged MFI for ICAM‐1, P‐selectin, E‐selectin, and VCAM‐1 for control HPAECs and for HPAECs treated with 30 nM GSK101 for 24 hr. Data on graphs are expressed as means ± *SEM* and represent the average of 17 experiments. Statistical analysis was performed using *t* test (***p < .001). (c) Averaged mean MFI for ICAM‐1 in control HUVECs (non‐treated), in HUVECs stimulated with 30 nM GSK101, and in GSK101‐stimulated HUVECs pre‐treated and incubated with TRPV4 antagonists (HC06 (1 µM) or RN98 (10 µM)) or BAPTA‐AM (10 µM). Data on graphs are expressed as means ± *SEM* and represent the average of eight experiments. Statistical analysis was performed using 1‐way ANOVA (**p < .01, ***p < .001). (d) Averaged mean MFI for ICAM‐1 in control HPAECs (non‐treated), in HPAECs stimulated with 30 nM GSK101, and in GSK101‐stimulated HPAECs pre‐treated and incubated with TRPV4 antagonists (HC06 (1 µM) or RN98 (10 µM)), or BAPTA‐AM (30 µM). Data on graphs are expressed as means ± *SEM* and represent the average of five experiments. Statistical analysis was performed using 1‐way ANOVA (**p < .01, ***p < .001)

**Figure 6:**
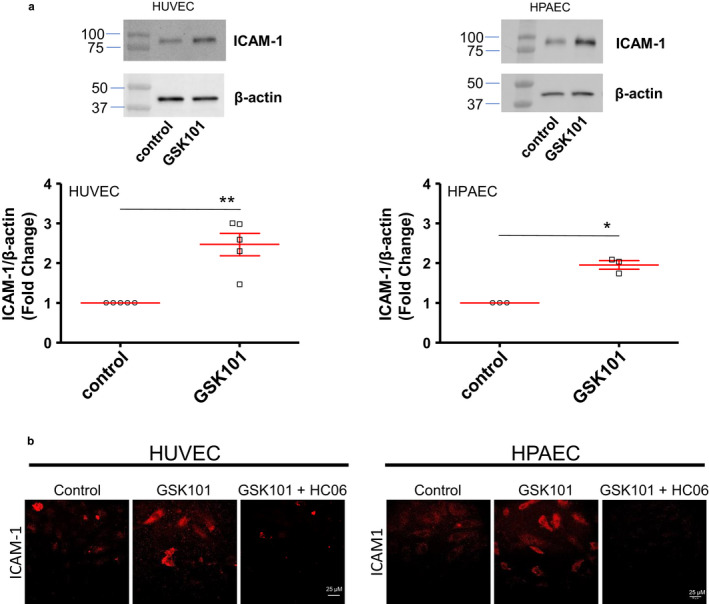
Effect of the TRPV4 agonist GSK101 on ICAM‐1 expression and localization in HUVECs and HPAECs. (a) Western Blot analysis of ICAM‐1 in control cells (HUVECs or HPAECs) and in cells stimulated with GSK101 (30 nM) for 24 hr. Graphs represent average values of five and three experiments for HUVECs and HPAECs, respectively (*p < .05, **p < .01). (b) Immuno‐localization of ICAM‐1 in HUVEC or HPAEC monolayers in control and after stimulation with GSK101 for 24 hr (with or without pre‐treatment with HC06 (1 µM)). Data presented are the representative of four experiments

**Figure 7:**
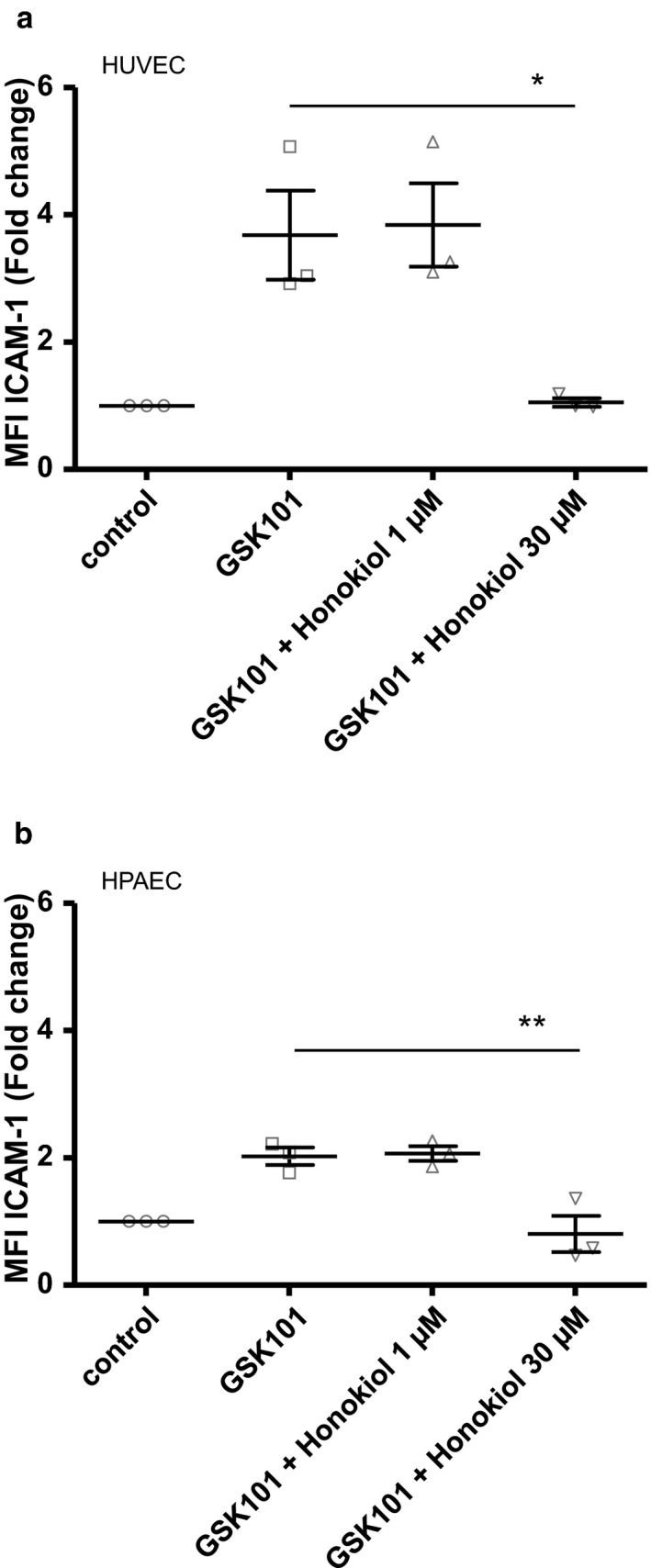
Transcription factors involved in GSK101‐induced effect on ICAM‐1 surface expression. Surface expression of ICAM‐1 was determined by flow cytometry 24 hr after the addition of 30 nM GSK101 to the culture medium of HUVECs or HPAECs. (a) Averaged MFI of ICAM‐1 for non‐treated HUVECs, for HUVECs stimulated with 30 nM GSK101, and for GSK101‐stimulated HUVECs pre‐treated and incubated with Honokiol [1 and 30 µM]). (b) Averaged MFI of ICAM‐1 for non‐treated HPAECs, for HPAECs stimulated with 30 nM GSK101, and for GSK101‐stimulated HPAECs pre‐treated and incubated with Honokiol (1 and 30 µM)). Data on graphs are expressed as means ± *SEM* and represent the average of three independent experiments. Statistical analysis was performed using 1‐way ANOVA (*p < .05, **p < .01)

**Figure 8:**
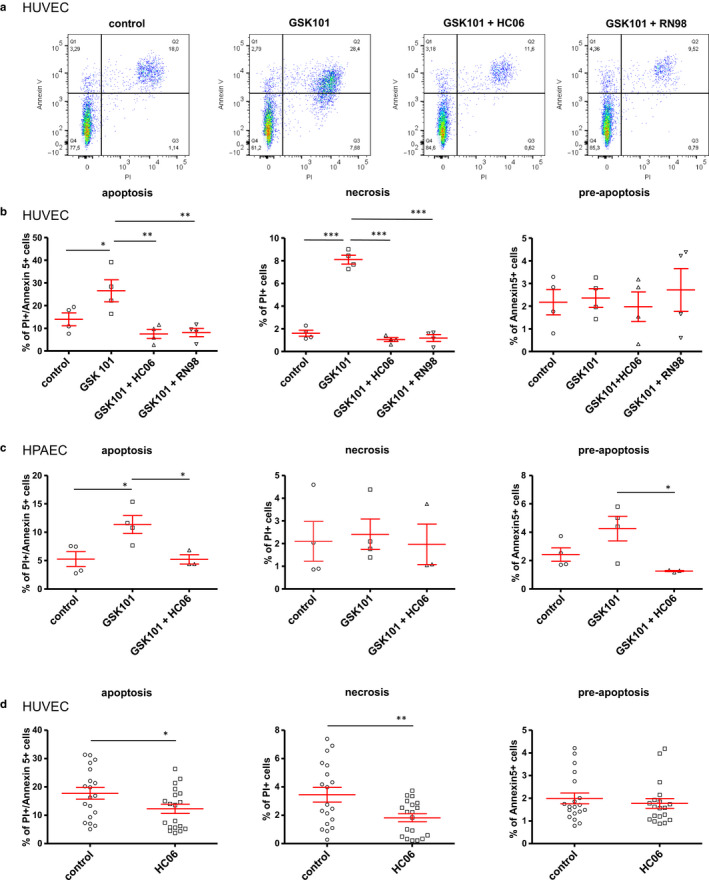
Effect of GSK101 and HC06 on apoptosis and necrosis in HUVECs and HPAECs. (a) Flow cytometry analysis of HUVECs in control and after incubation with GSK101 (for 24 hr) with or without TRPV4 antagonists (HC06 (1 µM) or RN98 (10 µM)). Quadrants drawn on histograms allow to define the repartition of HUVEC populations among apoptotic (Annexin V+/PI+), necrotic (Annexin V‐/PI+), pre‐apoptotic (Annexin V+/PI‐), and normal (Annexin V‐/PI‐) cells. (b) Mean averaged proportion or apoptotic (Annexin V+/PI+), necrotic (Annexin V‐/PI+), and pre‐apoptotic (Annexin V+/PI‐) cells among HUVEC populations for each condition. Data on graphs are expressed as means ± *SEM* and represent the average of four independent experiments. Statistical analysis was performed using 1‐way ANOVA (* *p* < .05, ** *p* < .01, *** *p* < .001). (c) Mean averaged proportion or apoptotic (Annexin V+/PI+), necrotic (Annexin V‐/PI+), and pre‐apoptotic (Annexin V+/PI‐) cells among HPAEC populations in control and after treatment with GSK101, with or without preincubation with HC06 (1 µM). Data on graphs are expressed as means ± *SEM* and represent the average of four independent experiments. Statistical analysis was performed using 1‐way ANOVA (* *p* < .05). (d) Effect of HC06 on basal/natural apoptosis of HUVECs. Apoptosis of HUVECs was measured by flow cytometry 48 hr after changing the culture medium, with or without incubation with 1µM HC06. Graphs represent the mean averaged proportion of apoptotic (Annexin V+/PI+), necrotic (Annexin V‐/PI+) and pre‐apoptotic (Annexin V+/PI‐) cells among HUVEC populations in control and in cells incubated with HC06. Data on graphs are expressed as means ± *SEM* and represent the average of data from six experiments (19 wells for each condition). Statistical analysis was performed using *t*‐test (* *p* < .05, ** *p* < .01)

**Figure 9:**
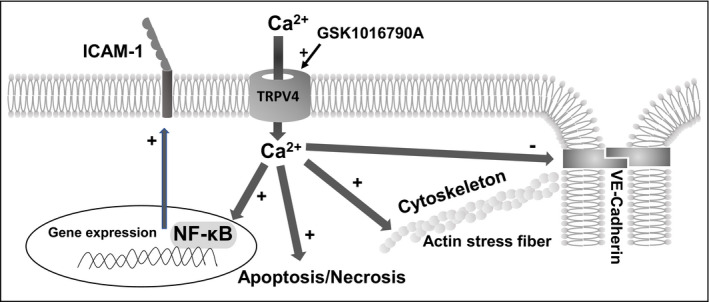
Multiple pro‐inflammatory effects of Ca^2+^ entry through TRPV4 channels in venous and arterial endothelial cells. TRPV4 channel opening triggers cytoskeleton rearrangement/actin stress fiber formation, downregulation of adherent junction made of VE‐Cadherin and increased endothelial permeability, enhanced membrane expression of the adhesion molecule ICAM‐1, and apoptosis/necrosis. Therefore, Ca^2+^ influx through TRPV4 channels promotes the transition of both venous and arterial endothelial cells toward a pro‐inflammatory phenotype

While triggering actin stress fiber formation, TRPV4 stimulation with GSK101 for 24 hr induced significant plasma membrane VE‐Cadherin downregulation in HUVECs and HPAECs, as evidenced by the decrease of plasma membrane‐associated fluorescence intensity for VE‐Cadherin (Figures 3, 4, 5, 6, 7, 8, 9). Plasma membrane VE‐Cadherin staining became thinner and rather discontinuous after GSK101 treatment in both HUVEC and HPAEC monolayers (Figure 3b). In contrast, VE‐Cadherin was significantly increased in the nucleus and cytosol of both HUVECs and HPAECs after GSK101 treatment (Figure 3b,c). The effect of TRPV4 activation on VE‐Cadherin localization was completely blocked when cells had been pre‐incubated with HC06 (Figure 3a‐c). Altogether this indicates that TRPV4 activation triggers the destabilization/delocalization of adherens junctions made of VE‐Cadherin. Intracellular increases of VE‐Cadherin observed in the cytosol also suggest that VE‐Cadherin may have been internalized.

### 3.4 TRPV4 activation decreased trans‐endothelial electrical resistance in both venous and arterial endothelial cells

In confluent HUVEC and HPAEC monolayers, TRPV4 stimulation with GSK101 (30 nM) quickly (less than 30 min) and strongly reduced endothelial monolayer impedance/electrical resistance, indicating that the activation of the channel alters monolayer barrier integrity (Figure 4a,b). This effect of TRPV4 activation was partly reversible and completely abolished when cells had been pre‐incubated with the TRPV4 channel inhibitor HC06 (1 µM). However, the effect of TRPV4 activation on endothelial monolayer electrical resistance was weaker in HPAEC monolayers.

### 3.5 TRPV4 activation increases the surface expression of ICAM‐1 in both venous and arterial endothelial cells

Confluent HUVECs or HPAECs were incubated for 24 hr with GSK101 (30 nM) alone or in combination with TRPV4 inhibitors (HC06 (1µM) or RN98 (10 µM)). Flow cytometry analysis (4 color) was then performed to assess membrane expression levels of adhesion molecules (ICAM‐1, VCAM‐1, E‐selectin, P‐selectin). After 24 hr incubation with GSK101, membrane expression levels of ICAM‐1 were found to be significantly increased when compared to unstimulated cells in both HUVECs and HPAECs, as evidenced by significant increases in ICAM‐1 MFI (Figure 5a,b). Inversely to what was observed for ICAM‐1, membrane expression levels of other adhesion molecules (VCAM‐1, P‐selectin, and E‐selectin) were not affected after 24 hr incubation with GSK101 (Figure 5a,b). Similar effects were observed when HUVECs or HPAECs were stimulated with another TRPV4 activator (RN 1747, not shown), but this TRPV4 agonist was much less potent than GSK101, as effects on ICAM‐1 membrane expression were only observed at concentrations ≥10 µM.

In both HUVECs and HPAECs, GSK101‐induced ICAM‐1 increases were strongly inhibited when cells had been preincubated with HC06 (1 µM) or RN98 (10 µM), indicating that these effects are, indeed, related to TRPV4 activation by GSK101, but not to another non‐specific effect of this agonist (Figure 5c,d). Preincubation of HUVECs or HPAECs with the membrane‐permeant intracellular Ca^2+^ chelator BAPTA‐AM significantly reduced GSK101‐induced ICAM‐1 overexpression, indicating that GSK101‐induced ICAM‐1 expression is a Ca^2+^‐dependent process (Figure 5c,d). While 30 µM BAPTA‐AM was used in HPAECs, HUVECs were pre‐treated with only 10 µM of this Ca^2+^ chelator, to avoid deleterious effects observed with high BAPTA‐AM concentrations in these cells. Therefore, the higher BAPTA‐AM concentration used in HPAECs may explain the larger inhibitory effect observed on GSK101‐induced ICAM‐1 expression in these cells (Figure 5c,d). Altogether, these results suggest that GSK101 induced Ca^2+^ entry through TRPV4 channels, resulting in increased membrane expression of ICAM‐1 in both HUVECs and HPAECs.

Our flow cytometry data indicate that the preincubation of venous or arterial endothelial cells with GSK101 increased ICAM‐1 membrane expression. To investigate whether these effects are related to protein synthesis and/or increased plasma membrane localization of ICAM‐1, we performed western blot analysis of ICAM‐1 expression in both HUVECs and HPAECs after 24 hr incubation with GSK101. In accordance with flow cytometry data, ICAM‐1 expression levels were found to be increased in both HUVECs and HPAECs after 24 hr incubation with GSK101, when compared to unstimulated cell (Figure 6a). Increased membrane expression levels of ICAM‐1 after TRPV4 channel activation was also confirmed by immunocytochemistry (Figure 6b). Moreover, increases in ICAM‐1 MFI were not observed when cells had been incubated for only 30 min with GSK101 (not shown). Altogether, these results indicate that TRPV4 stimulation with GSK101 increases the membrane expression of ICAM‐1, through a de novo synthesis of the protein.

### 3.6 Transcription factor involved in TRPV4 activation‐induced ICAM‐1 expression

In HUVECS, ICAM‐1 gene expression has been previously demonstrated to be regulated by the NF‐κB transcription factor downstream of Protease‐activated receptor 1 (PAR‐1) activation by thrombin (Xue et al., 2009). Honokiol and Ro106‐9920 have been proved to be potent inhibitors of NF‐κB activity (Swinney et al., 2002; Tse et al., 2005). As shown in Figure 7a,b, preincubating either HUVECs or HPAECs with the NF‐κB inhibitor Honokiol (30 µM) abolished the GSK101 effect. Similar effects were observed with another inhibitor of NF‐κB activity (Ro106‐9920, not shown). Therefore, NF‐κB is likely a key transcription factor involved in the stimulation of ICAM‐1 expression following stimulation by GSK101.

### 3.7 Induction of apoptosis upon TRPV4 activation in both venous and arterial endothelial cells

Confluent HUVECs were incubated for 24 hr with GSK101 (30 nM) alone or in combination with TRPV4 inhibitors (HC06 (1 µM) or RN98 (10 µM). Flow cytometry and an Annexin V‐FITC/ Propidium iodide (PI) apoptosis assay kit were used to measure the % of apoptotic (Annexin V+/PI+), necrotic (Annexin V‐/PI+) or pre‐apoptotic (Annexin V+/PI‐) cells among populations. Incubation for 24 hr with GSK101 (30 nM) significantly increased the fraction of apoptotic cells (Annexin V+/PI+) in HUVEC cultures, when compared to controls (Figure 8a,b). GSK101‐induced apoptosis was completely blocked when cells had been preincubated with either HC06 (1 µM) or RN98 (10 µM), showing that this effect relies on TRPV4 activation, rather than another non‐specific action on endothelial cells (Figure 8a,b). In addition to apoptosis, incubation with GSK101 also promoted necrosis for a limited fraction of cells (Annexin V‐/PI+), and similarly, the necrotic effect of GSK101 was blunted when TRPV4 channels were blocked by preincubation with HC06 (1 µM) or RN9893 (10 µM) (Figure 8a,b). In contrast, the small fraction of pre‐apoptotic cells observed in control conditions was not increased after TRPV4 stimulation with GSK101 (Figure 8a,b). As shown for HUVECs, treatment of HPAEC cultures with GSK101 for 24 hr triggered apoptosis that was inhibited by HC06 (Figure 8c). Treatment with HC06 also reduced the fraction of pre‐apoptotic cells (Annexin V+/PI‐). However, GSK101‐induced apoptosis was rather modest in HPAECs after 24 hr and in contrast to HUVECs, necrosis was not induced (Figure 8c).

### 3.8 Effect of TRPV4 blockade on natural apoptosis in endothelial cell cultures

Interestingly, we observed that preincubation with either HC06 or RN98 decreased the percentage of apoptotic cells in response to GSK101 to a lower level than that observed in control unstimulated cells, suggesting a role for TRPV4 opening in basal apoptosis (Figure 8a,b). To confirm this observation, control basal/natural apoptosis was measured in confluent HUVECs 48 hr after medium replacement, using the Annexin V‐FITC/ Propidium iodide (PI) apoptosis assay kit (Figure 8d). Apoptosis measurements in endothelial cultures were performed in control conditions or in the continuous presence of HC06 (1µM). Basal/ natural apoptosis and necrosis occurring spontaneously in confluent HUVEC cultures were found to be significantly reduced when cells had been preincubated with the TRPV4 channel blocker HC06 (Figure 8d).

## 4 DISCUSSION

In this study, we investigated the role of TRPV4 channels in pro‐inflammatory property development in both venous (HUVEC) and arterial (HPAEC) endothelial cells.

TRPV4 channels are robustly expressed in endothelial cells and as a consequence, stimulation of confluent monolayers with the potent TRPV4 agonist GSK101 triggered sustained cytosolic Ca^2+^ increases in both HUVECs and HPAECs, as previously demonstrated in HUVECs (Baratchi et al., 2019). GSK101‐induced endothelial Ca^2+^ responses were strongly reduced when cells had been preincubated with potent TRPV4 inhibitors (HC06 or RN98), indicating that these responses rely on the TRPV4 channel activation rather than on a non‐specific effect of the drug. Moreover, GSK101‐induced Ca^2+^ responses were abolished in the absence of external Ca^2+^. Altogether these results suggest that recorded GSK101‐induced Ca^2+^ responses are at least initiated by Ca^2+^ entry through TRPV4 channels, which does not preclude possible involvement of Ca^2+^ release from the sarcoplasmic reticulum (Shen et al., 2019).

In line with studies performed in other models, TRPV4 activation with GSK101 triggered rearrangement of the actin cytoskeleton and adherens junctions in both venous and arterial endothelial cells (Matsumoto et al., 2018; Phuong et al., 2017; Ryskamp et al., 2016; Zhao et al., 2018). Enhanced formation of F‐actin stress fiber was, indeed, observed following TRPV4 stimulation, while adherens junctions composed of VE‐cadherin were found to be downregulated. This enhanced formation of F‐actin stress fiber is likely linked to TRPV4‐induced Ca^2+^ entry, as increases in cytosolic Ca^2+^ through TRP channels or intracellular release have been demonstrated to activate both Ca^2+^‐calmodulin (CaM)‐dependent myosin light‐chain kinase (MLCK), protein kinase Cα (PKCα) and RhoA/ROCK pathways, leading to MLC phosphorylation, cytoskeletal rearrangement and stress fiber formation (De et al., 2013; Ryskamp et al., 2016; Weber & Muller, 2017). The observed downregulation of adherens junctions (plasma membrane VE‐Cadherin) may be partly linked to the apparition of these stress fibers, which can, indeed, exert tension on adherens junctions, promoting VE‐Cadherin destabilization/disassembly and internalization (Komarova et al., 2017; Weber & Muller, 2017). However, disassembly/destabilization and internalization of VE‐Cadherin may also be linked to its phosphorylation state (Komarova et al., 2017). While plasma membrane VE‐cadherin was downregulated, intracellular VE‐Cadherin was significantly increased, suggesting that the protein may have been internalized. Alternatively, this increase may correspond to a de novo synthesis. In addition to this long‐term downregulation of adherens junctions, TRPV4 channel activation quickly decreased endothelial monolayer electrical resistance of HUVEC and HPAEC monolayers, indicating rapid loss of barrier integrity, in accordance with previous reports (Phuong et al., 2017; Thorneloe et al., 2012). Decreases in endothelial monolayer electrical resistance were rather transient (partly reversible within 1 hr), suggesting that TRPV4 activation decreases endothelial monolayer electrical resistance through reversible junction destabilization (Phuong et al., 2017). Therefore, TRPV4 channel activation seems to exert both short‐term (destabilization) and long‐term (downregulation) effects on endothelial junctions.

In endothelial cells as well as in other cell types, cytosolic Ca^2+^ increases can govern gene expression through the activation of Ca^2+^‐dependent transcription factors. In lung endothelial cells, it has been demonstrated that Ca^2+^ entry through voltage‐dependent T‐type Ca^2+^ channel (α1G) or TRPV4 channels increase P‐selectin surface expression (Bihari et al., 2017; Wu et al., 2009). The role of TRPV4 channels in the regulation of eNOS expression has also been described in brain microvascular endothelial cells (Pu et al., 2015). Here, we show that the pharmacological activation of TRPV4 channels selectively increased ICAM‐1 surface expression in both arterial and venous endothelial cells, but was without significant effect on surface expression levels of other adhesion molecules including P‐selectin. Western blot analysis indicates that the increased ICAM‐1 surface expression observed in HUVECs and HPAECs can be explained by increased transcription of the ICAM‐1 gene. Increased ICAM‐1 expression has been demonstrated to increase RhoA and to exert positive feedback in actin rearrangement (Cerutti & Ridley, 2017; Thompson et al., 2002). In our study, the observed rise in ICAM‐1 expression may, therefore, further contribute to the cytoskeleton rearrangement and alteration of the endothelial barrier.

The ICAM‐1 gene promoter is mainly controlled by the transcription factor NF‐κB when HUVECs were activated by thrombin (Xue et al., 2009). Our results indicate that NF‐κB is also the transcription factor involved in the effect of GSK101 on the ICAM‐1 surface expression level. Overall, our results establish a link between Ca^2+^ signals induced by TRPV4 activation and ICAM‐1 surface expression in both venous and arterial endothelial cells, through NF‐κB activation, suggesting that it may be a general mechanism inducing ICAM‐1 expression in the whole vasculature. A similar mechanism of NF‐κB activation and ICAM‐1/VCAM‐1 expression has been recently reported following Ca^2+^ entry through TRP channels from the TRPC family (TRPC6, TRPC1, and TRPC3 channels) (Bodiga et al., 2015; Chen et al., 2019; Smedlund et al., 2010).

While endothelial P‐ and E‐selectin are rather involved in the rolling of neutrophils or leukocytes on the endothelial monolayer, ICAM‐1 and VCAM‐1 exert prominent roles in diapedesis, as they bind leukocyte/neutrophil integrins, allowing strong leukocyte/neutrophil adhesion to endothelial cells and further migration of inflammatory cells through the endothelium (Filippi, 2016; Langer & Chavakis, 2009; Lyck & Enzmann, 2015; McEver, 2015). TRPV4 channel activity, by controlling ICAM‐1 expression, may, therefore, appear as a regulator of leukocyte/neutrophil adhesion to the vascular endothelium. Moreover, TRPV4 activation enhanced ICAM‐1 expression in both venous and arterial endothelial cells, suggesting that this may represent a general mechanism of endothelial ICAM‐1 expression regulation throughout the whole vasculature, as TRPV4 channels are expressed in most, if not all endothelial cells (Earley & Brayden, 2015; White et al., 2016). In resting endothelial cells, ICAM‐1 membrane expression is very weak, but it increases dramatically in the case of endothelial injury, which is one of the major features of endothelial dysfunction occurring in numerous pathological states such as atherosclerosis, heart failure, hypertension, diabetes or septicemia (Deanfield et al., 2007; Kwan et al., 2007; Liao, 2013; Sena et al., 2013). In pathological states involving an ICAM‐1 overexpression on the endothelium, TRPV4 channels may then appear as an interesting target to reduce endothelial ICAM‐1 expression, leukocyte adhesion, and infiltration of inflammatory cells into tissues.

We also show here that GSK101‐induced TRPV4 activation triggers mainly apoptosis and few necrosis in venous and arterial endothelial cells. GSK101‐induced apoptosis was, indeed, prevented when endothelial cells had been preincubated with TRPV4 inhibitors. Apoptosis linked to TRPV4 channel‐induced Ca^2+^ entry has been described in other cell types including neurons, keratinocytes, and melanoma cells (Jie, et al., 2015; Olivan‐Viguera et al., 2018). In our models, treatment with HC06 prevented GSK101‐induced apoptosis and even reduced the fraction of apoptotic cells to lower levels than non‐treated/control cells. Moreover, incubation of confluent venous endothelial cells with HC06 significantly reduced basal/natural apoptosis occurring in confluent primary cell cultures. These results indicate that the TRPV4 blockade can partly protect endothelial cells from apoptosis or necrosis. They also suggest that a basal TRPV4 channel activity participates in the onset of apoptosis and necrosis in endothelial cells.

Overall, in spite of similar TRPV4 expression in venous and arterial endothelial cells, TRPV4 activation triggered cellular responses of rather higher amplitude in HUVECs than in HPAECs (endothelial barrier alteration, ICAM‐1 membrane expression, and apoptosis), suggesting that the amplitude of cellular responses triggered by TRPV4 activation is not correlated with the amount of TRPV4 channels in endothelial cells.

To conclude, our results indicate that TRPV4 activation can induce the transition of both venous and arterial endothelial cells toward a pro‐inflammatory phenotype, as evidenced by altered barrier integrity, increased membrane expression levels of ICAM‐1 and apoptosis (Figure 9). Finally, these results suggest that TRPV4 activity may favor diapedesis and inflammation through concomitant effects on endothelial junctions (destabilization, downregulation) and increased membrane expression of ICAM‐1.

## CONFLICT OF INTEREST

The authors declare no conflicts of interest.

## AUTHOR CONTRIBUTIONS

F‐X.B., R.B., and K.B. conceived the experiments; K.B., F‐X.B., R.B, and F.S. performed the experiments; K.B., F‐X.B., F.R., and R.B analyzed and interpreted the data; K.B., F‐X.B., and R.B. prepared the figures; F‐X.B. drafted the manuscript; R.B., F‐X.B., K.B., D.B., and D.R. revised the manuscript; F‐X.B. edited the final version of the manuscript.
